# Early ultrasound-based assessment of preterm white matter injury: association with MRI and neurological outcomes

**DOI:** 10.1007/s00247-026-06528-y

**Published:** 2026-02-18

**Authors:** Janah May Oclaman, Felicia Tang, Natalie Chan, Katelin Kramer, Ari J. Green, Dawn Gano, Fei Jiang, Kayla Cort, Yi Li, Bridget Elaine LaMonica Ostrem

**Affiliations:** 1 Department of Neurology, University of California, San Francisco, 675 Nelson Rising Lane, San Francisco, CA 94158 USA; 2 Department of Radiology, University of California, San Francisco, 505 Parnassus Ave, San Francisco, CA 94143 USA; 3 Department of Pediatrics, University of California, San Francisco, San Francisco, USA; 4 Department of Pediatrics, University of British Columbia, Vancouver, Canada; 5 Department of Epidemiology and Biostatistics, University of California, San Francisco, San Francisco, USA

**Keywords:** Premature infant, White matter, Neuroimaging, Cerebral palsy

## Abstract

**Background:**

White matter injury is a leading cause of neurodevelopmental impairment in premature infants. Timely initiation of novel therapies in clinical development will require early identification of clinically significant white matter injury. Head ultrasound is commonly obtained at 7 days and 30 days of life (DOL) to screen for brain injuries in premature infants.

**Objective:**

To determine if white matter injury severity on head ultrasound at 7 DOL and 30 DOL is associated with white matter injury severity on term-equivalent age MRI and with neurological outcomes through age 2 years.

**Materials and methods:**

We identified subjects via a search of the electronic health record for preterm infants born at ≤32 weeks gestational age (GA) with evidence of white matter injury in neuroimaging reports. Head ultrasounds at 7 days and 30 days and term-equivalent age MRIs were scored using established scoring systems by three expert readers, with final scoring established by consensus. We used ordinal logistic regression to determine the association between white matter severity on ultrasound and MRI. Multivariable models were adjusted for GA at birth and severity of intraventricular hemorrhage. Neurological outcomes (cerebral palsy, epilepsy, and neurosensory impairment) were determined by medical records review with a median corrected age at follow-up of 23.0 months.

**Results:**

Fifty infants with a median GA at birth of 27.1 weeks were included in our retrospective cohort. White matter injury severity on 7-DOL (odds ratio 1.8, 95% CI 1.3-2.6) and 30-DOL (odds ratio 1.5, 95% CI 1.2-2.0) ultrasound was independently associated with severity on MRI. Higher injury severity on 7-DOL ultrasound was associated with cerebral palsy (odds ratio 2.4, 95% CI 1.3-4.3), while higher injury severity on 30-DOL ultrasound was associated with both cerebral palsy (odds ratio 1.7, 95% CI 1.2-2.5) and neurosensory impairment (odds ratio 1.7, 95% CI 1.2-2.4).

**Conclusion:**

Preterm infants with white matter injury on 7-DOL or 30-DOL head ultrasound are at elevated risk for white matter injury on term-equivalent age brain MRI and for future neurological impairment.

**Graphical abstract:**

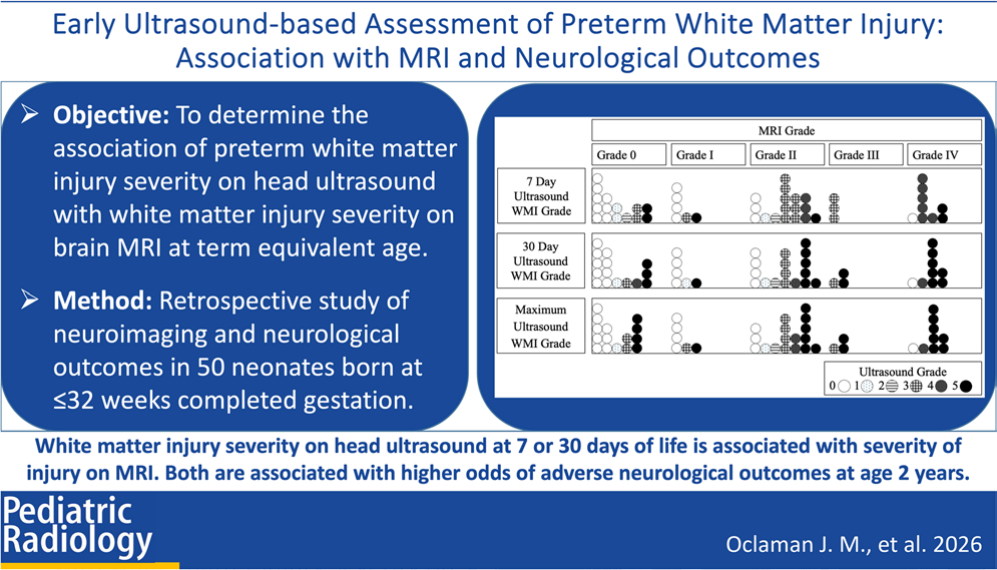

**Supplementary Information:**

The online version contains supplementary material available at 10.1007/s00247-026-06528-y.

## Introduction

Premature infants experience high rates of cognitive, language, visual, hearing, and motor impairments, and have a higher risk of epilepsy than babies born full-term [1–3]. Many of these adverse neurological outcomes are a consequence of white matter injury, the most common type of acquired brain injury in preterm infants [3–7]. While no targeted therapies currently exist for white matter injury, several neuroprotective and pro-myelinating compounds have shown promise in early clinical investigations and animal models of preterm brain injury [8–12]. Clinical trials of specific therapies for preterm white matter injury may soon commence, underscoring the need for early diagnosis of clinically-significant white matter injury by head ultrasound [13–15].

Head ultrasound can be performed at the bedside and can identify white matter injury early in the hospitalization of critically ill preterm neonates [16]. While brain MRI is considered superior to head ultrasound for diagnosis of preterm white matter injury, MRI requires specialized equipment, patient transport, and a degree of clinical stability often not present early in the hospitalization of very premature neonates [17]. MRI is therefore performed late in the hospitalization at most institutions, often at term-equivalent (near 40 weeks postmenstrual) age. In a recent study by Martinez-Biarge and colleagues, sequential head ultrasound was performed on preterm infants, followed by brain MRIs for patients who had been diagnosed with non-hemorrhagic white matter injury. A practical, reproducible MRI-based white matter injury scale was developed based on the relevant MRI findings [18]. The scale encompasses a range of white matter injury severity and is associated with future neurodevelopmental outcomes [18, 19]. However, the generalizability of this scale is unclear, as sequential ultrasounds are not commonly obtained in many intensive care nurseries. Many institutions follow the recommendations of the American Academy of Pediatrics, which include screening ultrasounds at 7 days and 30 days of life (DOL) in preterm infants, based primarily on the natural history of germinal matrix hemorrhage/intraventricular hemorrhage (GMH/IVH) and sequelae [20]. It is unknown whether white matter injury severity on head ultrasound at 7 DOL and 30 DOL predicts severity of white matter injury on term-equivalent age MRI on the Martinez-Biarge scale. The utility of the Martinez-Biarge scale in patients with concurrent GMH/IVH was also not extensively studied in the initial cohort [21].

The goal of the current study was to evaluate the association of white matter injury severity on head ultrasound at 7 DOL and 30 DOL with the severity of white matter injury on term-equivalent age MRI using the Martinez-Biarge scale. We used a retrospective, real-world cohort of preterm infants born at ≤ 32 weeks gestational age (GA) that included patients with a range of white matter injury and GMH/IVH severity. We hypothesized that white matter injury severity on head ultrasound at 7 DOL and 30 DOL would be associated with white matter injury severity on term-equivalent age MRI using the Martinez-Biarge scale, independent of GMH/IVH. We also hypothesized that white matter injury severity on head ultrasound and MRI would be associated with adverse neurological outcomes, including cerebral palsy, epilepsy, and neurosensory impairment.

## Materials and methods

### Project design, setting, and participants

**Fig. 1:**
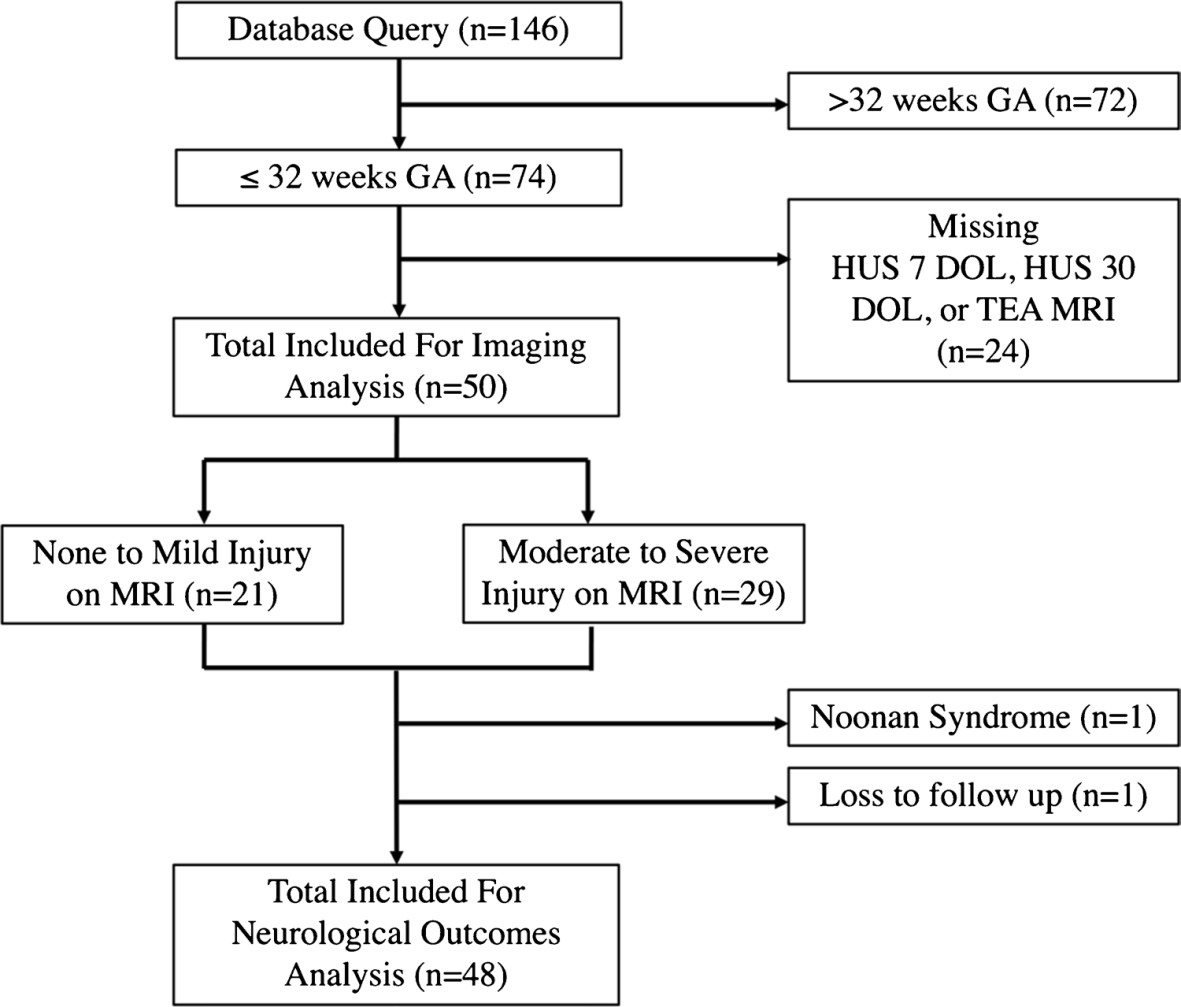
Study patient inclusion flowchart*.* No or mild white matter injury on MRI was defined as a Martinez-Biarge score of 0−1b. Moderate to severe white matter injury on MRI was defined as a Martinez-Biarge score of ≥2. *GA*, gestational age at birth; *HUS*, head ultrasound; *DOL*, days of life; *TEA*, term-equivalent age

This retrospective cohort study of infants previously hospitalized in one of two level IV neonatal intensive care units associated with a single institution was approved by our local institutional review board. Subjects were identified via a search of the medical record for patients born at ≤ 32 weeks completed gestation between January 1, 2016, and December 31, 2023, who were treated at one of two level IV neonatal intensive care units associated with a single institution, who were imaged at equal to or less than 6 months chronological age, and who had “white matter injury” or “periventricular leukomalacia” included in brain MRI or head ultrasound (head ultrasound) imaging reports. Each chart was reviewed manually to confirm availability of 7-DOL head ultrasound, 30-DOL head ultrasound, and term-equivalent age MRI scans for review. Of 146 potential subjects identified in the original database query, 96 were excluded due to GA at birth >32 weeks or absence of 7-DOL head ultrasound, 30-DOL head ultrasound, and/or term-equivalent age MRI that were available for review, resulting in 50 subjects included in this study (Fig. 1) (see Supplemental Methods for more information regarding subject selection and our institution’s standard screening protocol for preterm infants).

### Perinatal and neonatal data

Relevant clinical data were extracted from the electronic health record by manual chart review related to the neonatal hospitalization and outpatient clinic visits after hospital discharge. Neonatal and maternal demographics and medical history were collected from the delivery note, daily progress notes, discharge summary, medication administration record, and vitals flowsheets, and included GA at birth, birth weight, sex, prenatal steroid administration, surfactant administration, surgeries, and the following diagnoses if listed by the treating clinician in the medical record: maternal chorioamnionitis, necrotizing enterocolitis, patent ductus arteriosus, and bronchopulmonary dysplasia. Bronchopulmonary dysplasia diagnosis at our institution is diagnosed based on the level of respiratory support needed at 36 weeks postmenstrual age using NIH Consensus Criteria [22]. When possible, clinical variables were verified in at least two locations in the electronic health record.

### Head ultrasound and brain MRI review

Each participant’s 7-DOL and 30-DOL head ultrasound images were reviewed independently by two readers (reader 1 was a radiologist with 14 years of experience; reader 2 was a radiologist with 11 years of experience). White matter injury severity on head ultrasound was graded on a scale based on Miller 2003 criteria [23]. To understand the predictive value of maximum white matter injury severity on head ultrasound, we identified the higher white matter injury score between the 7-DOL and 30-DOL scans, referred to as the “maximum head ultrasound score.” Additional imaging factors assessed on head ultrasound included ventriculomegaly and germinal matrix hemorrhage/intraventricular hemorrhage (GMH/IVH), scored using the ordinal Papile grading system with the highest level of injury considered periventricular hemorrhagic infarction (PVHI) [23, 24]. Brain MRIs were acquired at term-equivalent age (median postmenstrual age [IQR]=39.1 [37.3, 41.6] weeks; range=34 2/7 weeks to 55 3/7 weeks) on a 3-T General Electric Discovery 750 (Waukesha, Wisconsin) or 1.5-T Philips Intera (Andover, MA) scanner. MRIs included the following sequences: diffusion-weighted imaging, T1- and T2-weighted sequences, and susceptibility-weighted imaging (sequence parameters are provided in Supplemental Table 1). MRIs were reviewed independently by two readers (reader 1 was a radiologist with 14 years of experience; reader 2 was a pediatric neurologist with 9 years of experience). White matter injury severity on MRI was graded based on the Martinez-Biarge 2016 criteria [18]. Additional imaging factors assessed on MRI included ventriculomegaly (defined by convention as atrial diameter greater than 10 mm) and GMH/IVH presence and severity [18]. For both imaging modalities, reviewers were blinded to the radiology report and clinical outcome, and consensus scores were determined by joint image review and discussion for studies that received discrepant scores in any category.

### Neurological outcomes

Neurological outcomes were available for 48 subjects in the cohort and were determined by electronic health record review of outpatient visit notes through the visit closest to 24 months corrected age (median [IQR] age at 2-year follow-up=23.9 [22.3, 25.9] months). Available clinic visits at older ages were also reviewed for information regarding neurological outcomes. Neurological outcomes (diagnosis of cerebral palsy (CP), diagnosis of epilepsy, and diagnosis of neurosensory (vision or hearing) impairment) were assessed independently by two reviewers blinded to the imaging results by examination of the medical record, with a consensus review performed for discrepant outcome determinations. Neurological outcome data were available for cerebral palsy in 39/48 (81%), neurosensory impairment in 41/48 (85%), and epilepsy in 48/48 (100%) of patients (see Supplemental Methods for more information regarding determination of neurological outcomes).

### Statistical analysis

Statistical analysis was performed using Stata version 17. Only patients with available data regarding each predictor and outcome variable were included in each analysis. Baseline characteristics were assessed in patients with mild or no (Martinez-Biarge grades 0−1) and with moderate to severe (Martinez-Biarge grades 2−4) white matter injury on term-equivalent age MRI using Student’s *t*-tests for parametric and Mann-Whitney tests for ordinal and non-parametric continuous variables (GA at birth, birth weight, child opportunity index, and intraventricular hemorrhage grade) and Fisher’s exact tests for binary variables (sex assigned at birth, prenatal steroids, maternal chorioamnionitis, surfactant, neonatal surgery, necrotizing enterocolitis, patent ductus arteriosus, GMH/IVH, and bronchopulmonary dysplasia).

The primary predictor was severity of white matter injury on head ultrasound based on the Miller 2003 criteria [23]. The primary outcome was severity of white matter injury on term-equivalent age MRI based on the Martinez-Biarge 2016 criteria [18]. Ordinal logistic regression was used to determine the association between the grade of white matter injury on ultrasound (7 DOL, 30 DOL, or “maximum” head ultrasound representing the higher score between 7 DOL and 30 DOL) and grade of white matter injury on MRI. Multivariable ordinal logistic regression included the following covariates: GA at birth and IVH grade on head ultrasound. Secondary endpoints included neurological outcomes at 2 years of age. We used logistic regression to determine the association between white matter injury severity on head ultrasound (7 DOL and 30 DOL) and MRI (term-equivalent age) with each neurological outcome. Multivariable models included the following covariates: gestational age at birth and IVH severity grade. For all analyses, a *P*-value <0.05 was considered statistically significant.

## Results

### Characteristics of the patient cohort

Subjects for the retrospective cohort were identified via a medical record search for patients born at ≤32 weeks completed gestation with evidence of white matter injury in neuroimaging reports. In total, 50 patients met inclusion criteria and had a 7-DOL head ultrasound, 30-DOL head ultrasound, and a term-equivalent age MRI available for review (Fig. 1). All 50 eligible patients were included in the study cohort. Baseline characteristics are reported in Table 1. Subjects were born at a median (IQR) age of 27.1 (25.1, 29.5) weeks. The majority of infants were exposed to prenatal steroids (86%), received surfactant (68%), and received a diagnosis of bronchopulmonary dysplasia (66%).

**Table 1:** Neonatal demographics and clinical characteristics

**Characteristics**	Study cohort (*N*=50)	No or mild white matter injury^a^ (*N*=21)	Moderate to severe white matter injury^a^ (*N*=29)	*P*-value^b^
**GA at birth, median weeks (IQR)**	27.1 (25.1, 29.5)	26.3 (25.1, 28.7)	27.1 (25, 29.6)	0.74
**Race, available *N***	41	17	24	0.66
Asian, *N* (%)	4 (9.6%)	1 (5.9%)	3 (12.5%)	
Black, *N* (%)	8 (19.5%)	2 (11.8%)	6 (25.0%)	
White, *N* (%)	20 (48.8%)	10 (58.8%)	10 (41.7%)	
Other, *N* (%)	9 (22.0%)	4 (23.5%)	5 (20.8%)	
**Male, *N* (%)**	27 (54.0%)	13 (61.9%)	14 (48.3%)	1.00
**Birth weight, median in grams (IQR)**	43 (86.0%)	935 (725, 1,164)	930 (695, 1,230)	0.82
**Prenatal steroids, *N* (%)**	5 (10.0%)	16 (76.2%)	27 (93.1%)	0.12
**Maternal chorioamnionitis, *N* (%)**	34 (68.0%)	2 (9.5%)	3 (10.3%)	1.00
**Received surfactant, *N* (%)**	30 (60.0%)	16 (76.2%)	18 (62.1%)	0.37
**Neonatal surgery, *N* (%)**	6 (12.0%)	14 (66.7%)	16 (55.2%)	0.56
**Necrotizing enterocolitis, *N* (%)**	23 (46.0%)	3 (14.3%)	3 (10.3%)	0.69
**Patent ductus arteriosus, *N* (%)**	33 (66.0%)	12 (57.1%)	11 (37.9%)	0.25
**Bronchopulmonary dysplasia, *N* (%)**	43 (86.0%)	13(61.9%)	20 (69.0%)	0.76
**Maximum IVH grade on head ultrasound**				** <0.01**
Grade 0, *N* (%)	17 (34.0%)	12 (57.1%)	5 (17.2%)	
Grade I, *N* (%)	10 (20.0%)	4 (19.1%)	6 (20.7%)	
Grade II, *N* (%)	12 (24.0%)	3 (14.3%)	9 (31.0%)	
Grade III, *N* (%)	0 (0.0%)	0 (0.0%)	0 (0.0%)	
PVHI, *N* (%)	11 (22.0%)	2 (9.5%)	9 (31.0%)	
**Postmenstrual age at term-equivalent age MRI, median weeks (IQR)**	39.1 (37.3, 41.6)	41.3 (37.1, 42.7)	38 (37.4, 40.1)	1.00
**Corrected age at 2-year follow-up, median months (IQR)**	23.0 (21.5, 25.0)	23.9 (22.3, 25.9)	22.9 (21.3, 24.8)	0.28

*IVH*, intraventricular hemorrhage; *CA*, corrected age; *PVHI*, periventricular hemorrhagic infarction

^a^Patient characteristics are shown in subjects with no or mild (grades 0-1b) compared to those with moderate to severe (grades 2–4) white matter injury on term-equivalent age MRI

^b^Binary variables were compared using Fisher’s exact test. Non-parametric continuous variables and ordinal variables were compared using a Mann-Whitney *U* test

### MRI and head ultrasound scoring

White matter injury severity was scored independently by two experienced raters followed by consensus review, using the Miller 2003 criteria for head ultrasounds and the Martinez-Biarge 2016 criteria for brain MRIs [18, 23] (Tables 2 and 3). Assessment of inter-rater reliability of white matter injury severity scores demonstrated a weighted kappa of 0.58, 0.64, and 0.73 for 7-DOL ultrasound, 30-DOL ultrasound, and term-equivalent age MRI, respectively. Patient characteristics were compared between subjects with no or mild white matter injury (Martinez-Biarge grades 0 to 1b) on MRI and moderate to severe white matter injury (Martinez-Biarge grades 2−4) on MRI (Table 1). The two groups did not differ significantly based on GA at birth or major maternal or neonatal comorbidities. Postmenstrual age at 7-DOL and 30-DOL head ultrasound, corrected age at term-equivalent age MRI, and corrected age at clinical follow-up did not differ significantly between groups. Patients with moderate to severe white matter injury on MRI had significantly higher GMH/IVH severity on head ultrasound (*P*<0.01, Mann-Whitney test).

**Table 2:** White matter injury severity grading criteria for head ultrasound

Grade	Description of white matter abnormalities	^a^*N*, 7 DOL	^a^*N*, 30 DOL
0	None	16	18
1	Small foci of abnormal echogenicity less echogenic than choroid plexus	3	3
2	Diffuse foci of increased echogenicity less than choroid plexus or irregularity of the lateral borders of the periventricular white matter (junction of the normal halo and surrounding cortex)	2	1
3	Focal areas of abnormal echogenicity greater than or equal to the echogenicity of the choroid plexus	14	6
4	Diffuse increased echogenicity greater than or equal to the echogenicity of the choroid plexus	9	3
5	Periventricular cysts (cavitation) defined as anechoic regions with increased through transmission	6	19

Table modified from Miller et al. (2003)

^a^*N*, number of subjects with each grade at 7 days and 30 days of life (*DOL*)

**Table 3:** White matter injury severity grading criteria for brain MRI

Grade	Description of white matter abnormalities	^a^*N*
**0**	None	15
**1a**	<6 punctate white matter lesions, focal increased signal intensity of the white matter on T1-weighted, symmetrical age appropriate or nearly age appropriate PLIC myelination	6
**1b**	≥6 punctate white matter lesions, focal increased signal intensity of the white matter on T1-weighted, symmetrical age appropriate or nearly age appropriate PLIC myelination	0
**2**	Focal periventricular cysts and/or at least 2 of the following: mild ventriculomegaly (7.5–10.5 mm at the atrium), irregularly shaped ventricles, few focal increased signal intensity white matter lesions on T1-weighted, sparse PLIC myelination	17
**3**	Extensive periventricular cysts, and/or at least 2 of the following: decreased white matter volume and mild-moderate ventriculomegaly (>10 mm), irregularly shaped ventricles, extensive increased signal intensity white matter lesion on T1-weighted, sparse or no PLIC myelination	3
4	Extensive periventricular and subcortical cysts, and/or at least 2 of the following: moderate-severe ventriculomegaly (>10 mm), severe-complete loss of white matter, basal ganglia/thalamic involvement, extensive increased signal intensity white matter lesions on T1-weighted imaging, no PLIC myelination	9

Table modified from Martinez-Biarge et al. (2016)

*PLIC*, posterior limb of the internal capsule

^a^*N*, number of subjects with each severity grade on term-equivalent age brain MRI

### Association of white matter injury severity on head ultrasound with white matter injury severity on term-equivalent age MRI

**Fig. 2:**
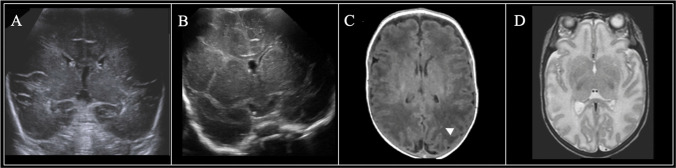
Neuroimaging findings in an infant with no evidence of white matter injury on head ultrasound. The male infant was born at 28 weeks and 5 days. Sequential imaging demonstrated (**A**) normal 7-DOL coronal-plane head ultrasound (Miller score 0); (**B**) normal 30-DOL coronal-plane head ultrasound (Miller score 0); and (**C**) term-equivalent MRI with a punctate focus of increased signal intensity in the white matter on T1-weighted imaging (*white arrowhead*, Martinez-Biarge score 1a). **D** Axial T2-weighted imaging was normal

**Fig. 3:**
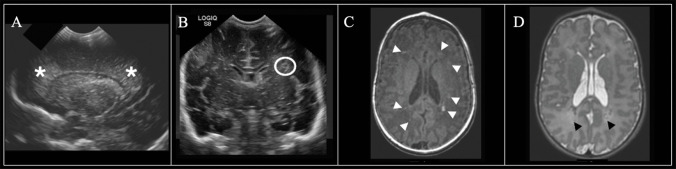
Sequential neuroimaging in a female infant born at 30 weeks. (**A**) 7-DOL coronal-plane head ultrasound demonstrated diffusely increased white matter echogenicity equal to or brighter than choroid plexus (*asterisk*, Miller score 4); (**B**) 30-DOL coronal-plane head ultrasound showed cavitary cystic change in the white matter (*white circles*, Miller score 5); term-equivalent age MRI demonstrated (**C**) greater than 6 foci of increased signal intensity in the white matter on axial T1-weighted imaging (*white arrowheads*) and (**D**) small periventricular cysts (*black arrow*) on axial T2-weighted imaging with decreased white matter volume (Martinez-Biarge score 2)

**Fig. 4:**
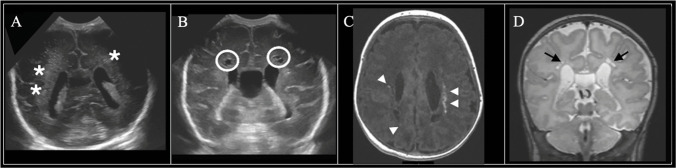
Sequential neuroimaging in a male infant born at 29 weeks and 5 days. (**A**) 7-DOL coronal-plane head ultrasound demonstrated focal areas of abnormal echogenicity greater than or equal to choroid plexus (*asterisk*, Miller score 3); (**B**) 30-DOL coronal-plane head ultrasound showed cavitary cystic lesions in the white matter (*white circles*, Miller score 5). Term-equivalent MRI demonstrated (**C**) greater than 6 foci of increased signal intensity in the white matter on axial T1-weighted imaging (*white arrowheads*) and (D) extensive periventricular cysts (*black arrow*) on axial T2-weighted imaging, with ventriculomegaly and decreased white matter volume (Martinez-Biarge score 3)

**Fig. 5:**
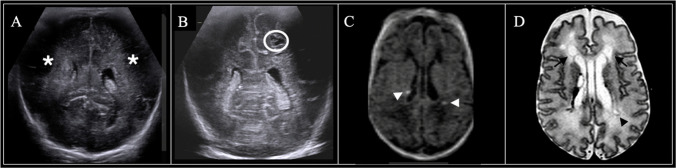
Neuroimaging in a female infant born at 29 weeks and 4 days with severe periventricular and subcortical cystic white matter injury. (**A**) 7-DOL coronal-plane head ultrasound demonstrated diffusely increased echogenicity equal to or brighter than choroid plexus (*asterisk*, Miller score 4); (**B**) 30-DOL coronal-plane head ultrasound demonstrated cavitary cystic lesions in the white matter (*white circles*, Miller score 5). Term-equivalent age MRI showed (**C**) greater than 6 foci of increased signal intensity in the white matter on axial T1-weighted imaging (*white arrowheads*, only a subset are marked for clarity) and (**D**) extensive periventricular and subcortical cysts (*black arrow*, only a subset are marked for clarity) and ventriculomegaly with decreased white matter volume. This patient also had grade II germinal matrix hemorrhage on head ultrasound (not shown), which evolved into germinolytic cysts on the term-equivalent age MRI. The Martinez-Biarge score was 4

**Fig. 6:**
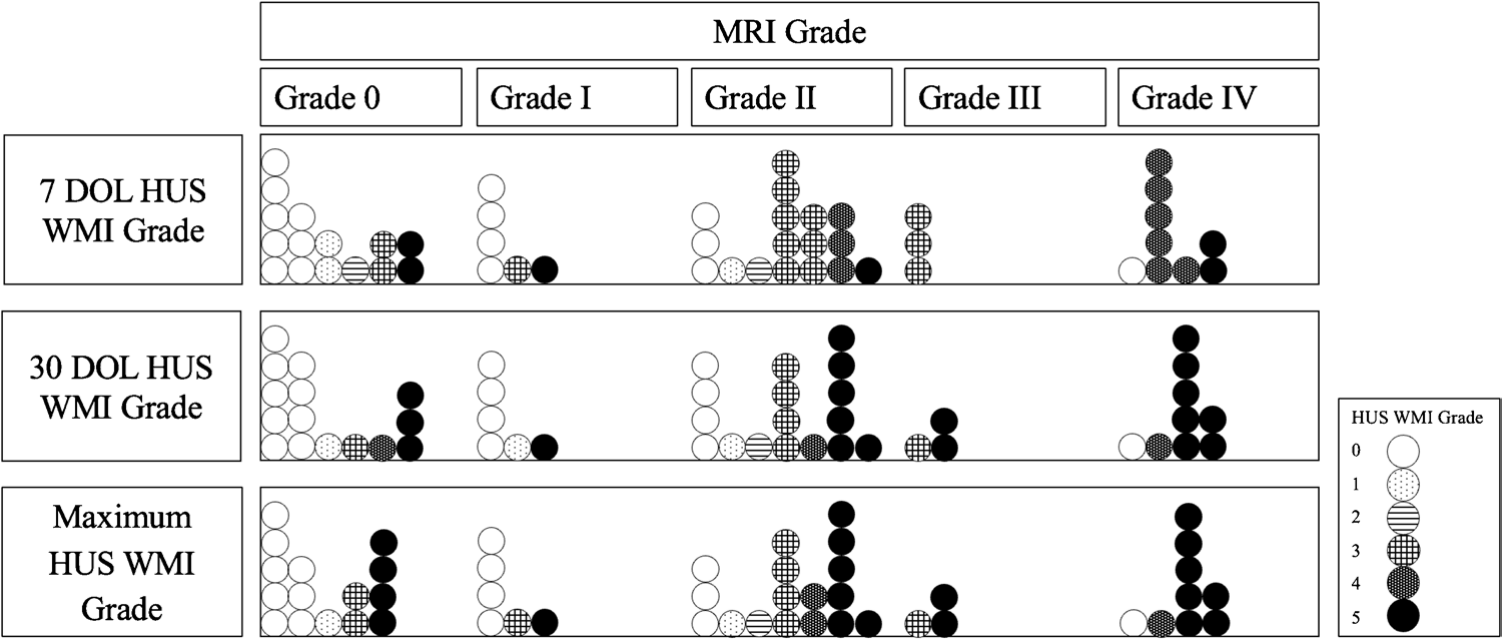
Evolution of white matter injury patterns in individual subjects between head ultrasound at 7 days and 30 days of life and term-equivalent age MRI. Each *circle* represents one subject. *Circles* are shaded based on white matter injury grade on head ultrasound. *DOL*, days of life; *HUS*, head ultrasound; *WMI*, white matter injury

Infants displayed a range of white matter injury severity on 7 DOL, 30 DOL, and term-equivalent age MRI (Figs. 2, 3, 4, and 5). Using ordinal logistic regression, we found that white matter injury severity on 7-DOL head ultrasound (OR=1.8, 95% CI 1.3–2.5) and 30-DOL head ultrasound (OR=1.6, 95% CI 1.2–2.1) was associated with higher odds of white matter injury on term-equivalent age MRI (Fig. 6, Table 4). Similar associations were observed in multivariable ordinal logistic models in which GA at birth and GMH/IVH severity were included as covariates (Table 4). Comparable results were also obtained in sensitivity analyses in which subjects with severe GMH/IVH or with cystic white matter injury were removed from the cohort (Supplemental Table 2). Thus, white matter injury severity on head ultrasound is independently associated with white matter injury severity on term-equivalent age MRI.

**Table 4:** Association of white matter injury severity on ultrasound with white matter injury severity on term-equivalent age MRI

Predictor	Increased white matter injury severity on MRI	
	OR (95% CI)	*P*-value
Univariable		
WMI severity, 7-DOL ultrasound	1.8 (1.3–2.5)	** <0.01**
WMI severity, 30-DOL ultrasound	1.6 (1.2–2.1)	** <0.01**
WMI severity, maximum ultrasound grade	1.6 (1.2–2.0)	** <0.01**
Multivariable		
WMI severity, 7-DOL ultrasound	1.8 (1.3–2.6)	** <0.01**
WMI severity, 30-DOL ultrasound	1.5 (1.2–2.0)	**0.01**
WMI severity, maximum ultrasound grade	1.4 (1.1–1.9)	** <0.01**

Multivariable models were adjusted for GA at birth and germinal matrix hemorrhage/intraventricular hemorrhage severity on the same scan (7-DOL head ultrasound, 30-DOL head ultrasound, or maximum between the 7-DOL and 30-DOL head ultrasounds)

*DOL* days of life, *OR* odds ratio, *CI* confidence interval

### Association of white matter injury severity with neurological outcomes

We tracked diagnoses of cerebral palsy (CP), epilepsy, and neurosensory (vision or hearing) impairment through 2 years of age (median [IQR] corrected age at 2-year follow-up was 23.0 [21.5, 25.0] months) [25, 26]. Outcomes were available for 39, 48, and 41 patients regarding CP, epilepsy, and neurosensory impairment, respectively. CP was diagnosed in 17/39 (44%), epilepsy in 8/48 (17%), and neurosensory impairment in 18/41 (44%) of patients. Using univariable logistic regression, we found that white matter injury severity on 7-DOL and 30-DOL head ultrasounds, maximum white matter injury grade on head ultrasound, and white matter injury grade on MRI were all associated with increased odds of CP (Table 5). In univariable models, white matter injury severity on MRI was associated with higher odds of epilepsy, while 30-DOL head ultrasound and maximum head ultrasound white matter injury severity were associated with increased odds of neurosensory impairment. Similar associations were found using multivariable models in which GA at birth and IVH severity on the same scan were included as covariates (Table 5).

**Table 5:** Association of white matter injury severity with neurological outcomes at 2 years of age

Predictor	Epilepsy *N*=48		Neurosensory impairment *N*=41		Cerebral palsy *N*=39	
	OR (95% CI)	*P*-value	OR (95% CI)	*P*-value	OR (95% CI)	*P*-value
Univariable						
WMI severity, 7-DOL ultrasound	1.4 (0.9–2.3)	0.16	1.4 (1.0–2.0)	0.08	2.4 (1.3–4.3)	**<0.01**
WMI severity, 30-DOL ultrasound	1.3 (0.9–1.9)	0.21	1.7 (1.2–2.4)	**<0.01**	1.7 (1.2–2.5)	**<0.01**
WMI severity, maximum ultrasound grade	1.2 (0.8–1.8)	0.31	1.6 (1.1–2.3)	**<0.01**	1.9 (1.2–2.9)	**<0.01**
WMI severity, MRI	6.0 (2.0–18.2)	**<0.01**	1.4 (0.9–2.1)	0.15	3.2 (1.6–6.5)	**<0.01**
Multivariable						
WMI severity, 7-DOL ultrasound	1.6 (0.8–3.3)	0.18	1.2 (0.8–1.9)	0.32	2.5 (1.3–4.9)	**<0.01**
WMI severity, 30-DOL ultrasound	1.4 (0.8–2.4)	0.29	1.6 (1.1–2.3)	**0.01**	1.6 (1.1–2.5)	**0.03**
WMI severity, maximum ultrasound grade	1.3 (0.8–2.2)	0.34	1.4 (1.1–2.1)	**0.05**	1.9 (1.1–3.0)	**0.01**
WMI severity, MRI	7.9 (1.7–36.5)	**<0.01**	1.4 (0.8–2.3)	0.28	3.2 (1.5–7.0)	** <0.01**

Multivariable models were adjusted for gestational age at birth and germinal matrix hemorrhage/intraventricular hemorrhage severity on the same scan

*OR* odds ratio, *CI* confidence interval, *WMI* white matter injury, *DOL* days of life

**Fig. 7:**
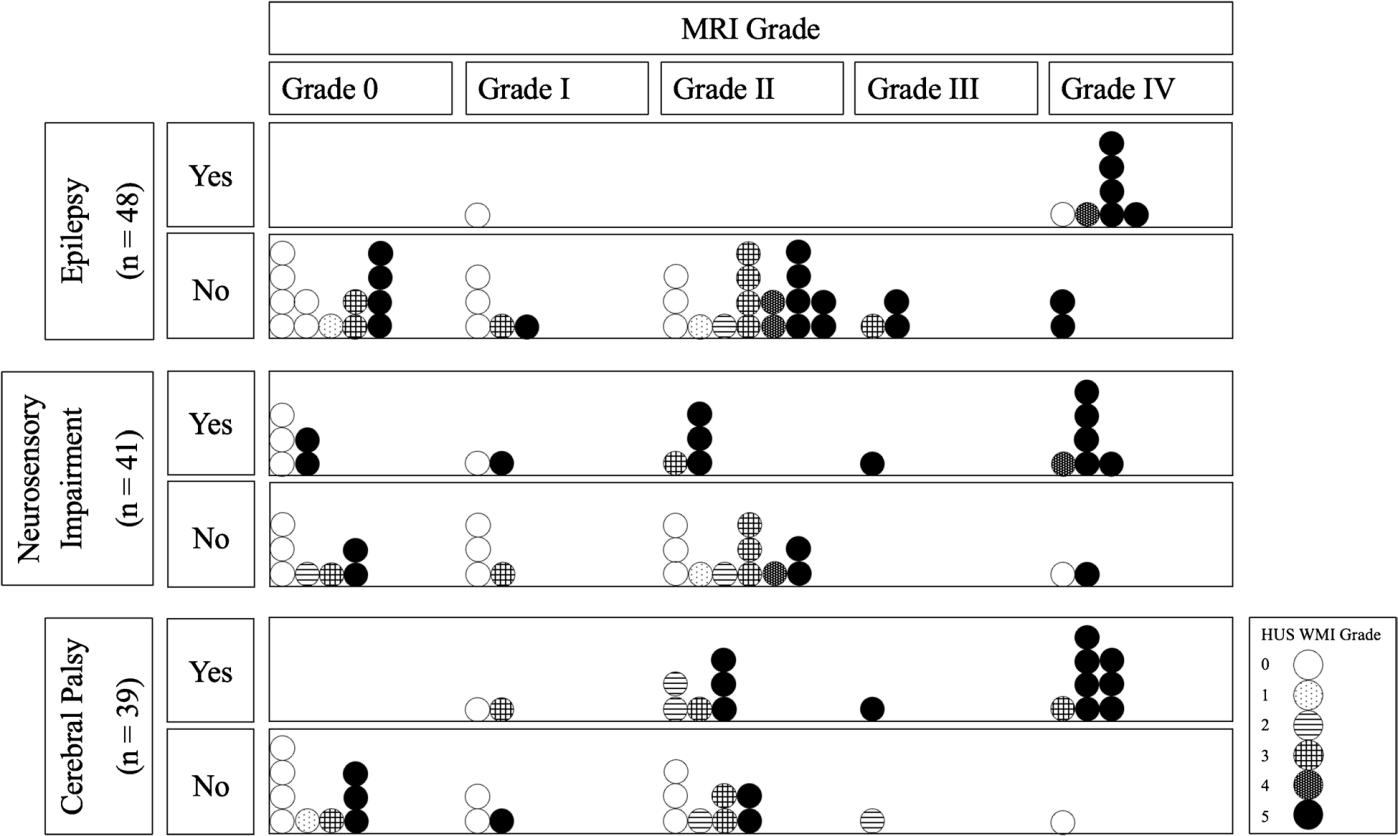
Neurological outcomes at 2 years of age. Neurological outcomes (epilepsy, cerebral palsy, and neurosensory impairment) are organized by white matter injury severity grade on term-equivalent age MRI. Each *circle* represents one subject. *Circles* are shaded based on maximum white matter injury grade between the 7-DOL and 30-DOL head ultrasounds. *HUS*, head ultrasound; *WMI*, white matter injury

The distribution of neurological outcomes by white matter injury severity on head ultrasound (maximum score) and MRI is shown in Fig. 7. The predictive value of each imaging modality for each outcome is shown in Table 6. For head ultrasounds, we examined a threshold Miller score of ≥3, as this level of injury has been reported to have a positive predictive value (PPV) of 100% for MRI abnormalities [23]. For brain MRIs, we examined a threshold Martinez-Biarge score of ≥2, the level of injury previously associated with high rates (5/6 patients (83.5%) in a small cohort) of CP [18]. Both imaging modalities showed higher PPV for CP than for epilepsy or neurosensory impairment.

**Table 6:** Predictive value of specific thresholds of white matter injury for adverse neurological outcomes

Neurological outcomes	Maximum head ultrasound grade ≥3		Term-equivalent age MRI grade ≥2	
	PPV	NPV	PPV	NPV
Epilepsy	19.4%	88.2%	24.1%	94.7%
Neurosensory impairment	56.0%	81.3%	45.8%	64.7%
Cerebral palsy	61.5%	92.3%	60.0%	85.7%

*PPV* positive predictive value, *NPV* negative predictive value

## Discussion

In this cohort of preterm infants born at equal to or less than 32 weeks, white matter injury severity on 7-DOL and 30-DOL head ultrasounds was associated with white matter injury severity on term-equivalent age MRI using the Martinez-Biarge grading system. This association was independent of the severity of GMH/IVH. Our findings support the validity of the scale developed by Martinez-Biarge and colleagues in preterm neonates with a range of white matter injury severity, and in patients with concurrent GMH/IVH.

We found that white matter injury severity on head ultrasound and MRI at all timepoints was independently associated with higher odds of CP at age 2 years. White matter injury severity on MRI, but not head ultrasound, was also associated with increased odds of epilepsy. These findings support the prognostic utility of both head ultrasound and MRI, while reinforcing prior evidence that MRI at term-equivalent age generally outperforms early head ultrasound in predicting neurological outcome [6, 8, 18, 27–30]. Interestingly, in our cohort, 30-DOL head ultrasound was independently associated with higher odds of neurosensory impairment, while 7-DOL head ultrasound and term-equivalent age MRI were not. This finding may be attributable to patients with cysts on head ultrasound (grade 5 injury) that had involuted by the time of term-equivalent age MRI, leading to lower white matter injury scores on MRI than on 30-DOL head ultrasound [18]. These observations highlight the dynamic nature of white matter injury, which can lead to disparate assessments of severity based on head ultrasound and MRI obtained at different time points, underscoring the importance of using both modalities in a complementary fashion. While our study suggests that clinically-significant white matter injury can be readily identified at 30 DOL, head ultrasound within the first week of life remains essential for identifying IVH/GMH and related complications, such as posthemorrhagic ventricular dilatation and hydrocephalus, that may require neurosurgical intervention [20].

In this study, we included infants with hemorrhagic injury given the common co-occurrence of different types of acquired brain injury in the preterm population. GMH/IVH may directly cause white matter injury through several mechanisms, including venous congestion and tissue infarction, white matter necrosis and cystic transformation after hemorrhage, and stretching of white matter tracts due to posthemorrhagic ventricular dilatation. Insults unrelated to GMH/IVH, such as hypoxia-ischemia, inflammation, and systemic or central nervous system infection, may also lead to white matter injury by inducing differentiation arrest and apoptosis of oligodendrocytes and oligodendrocyte precursors in the developing white matter [9, 31–33]. We controlled for GMH/IVH in multivariable models to better understand the association of white matter injury severity, regardless of cause, with neurological outcome [31]. We found that all associations between white matter injury severity and neurological outcome were independent of GMH/IVH severity. Our findings support the independent value of assessing white matter injury severity for predicting neurological outcome, whether white matter injury is related or unrelated to GMH/IVH.

Limitations of this study include its retrospective and single-institution design, which introduces the possibility of unmeasured confounders and limits generalizability. Patients were identified based on a search for white matter injury and similar terms in radiology reports, which introduces selection bias and limits the applicability of our findings to a broader population of preterm infants, particularly those without documented white matter injury. As patients with brain injury on head ultrasound are more likely to receive brain MRIs at our institution, this study was also subject to partial verification bias, which can overestimate sensitivity and underestimate specificity. Thus, we did not report sensitivity and specificity in this study, and instead reported PPV and NPV. The sickest patients may be underrepresented in this study, as some patients with very severe white matter injury on head ultrasound may be too unstable for timely term-equivalent age MRI or may die before the term-equivalent age is reached or before neurodevelopmental follow-up. This introduces survivor bias, which may underestimate the association between severe white matter injury on head ultrasound and later findings on term-equivalent age MRI. Furthermore, we reported on neurological outcomes available in the medical record, which were not collected as part of standardized assessments. This limited our ability to capture milder, but still clinically relevant, developmental delays or neurological symptoms. Finally, our analyses were limited by incomplete outcomes data for about 20% of infants and a relatively short follow-up duration (2 years).

The strengths of this study include a practical design based on a widely-employed screening imaging protocol for preterm infants, and a relatively large cohort with white matter injury compared to other similar studies [19]. We used head ultrasound and MRI severity scales that included gradations of diffuse, non-cystic white matter injury, reflective of the most common type of white matter injury seen in current clinical practice [18]. We demonstrated good inter-rater reliability validating the ease of use and wide applicability of these scoring systems. Overall, our findings support the use of both head ultrasound- and MRI-based inclusion criteria in upcoming clinical trials for preterm white matter injury. This approach will enable prompt treatment initiation to optimize brain recovery and neurological outcomes in this vulnerable population.

##  Supplementary Information

Below is the link to the electronic supplementary material.

ESM 1 (198 KB PDF)

ESM 2 (75.9 KB PDF)

## Data Availability

While identifiable patient data cannot be shared to protect study participant privacy, de-identified data generated during the current study are available from the contact authors on reasonable request.
