# Supplementary material for: Early ultrasound-based assessment of preterm white matter injury: association with MRI and neurological outcomes
Source: Pediatr Radiol. 2026 Feb 18;56(6):1308–19. doi: 10.1007/s00247-026-06528-y (PMC13212370; doi:10.1007/s00247-026-06528-y)
Supplement: Supplementary file 1 — (198 KB PDF) [file 247_2026_6528_MOESM1_ESM.pdf]

Supplementary Data

Supplemental Table 1. Brain MRI sequences and imaging parameters

	Details	TR (ms)	TE (ms)	TI (ms)	Echo Train Length	Flip angle	Receiver bandwidth	Slice/skip (mm)	FOV (cm)
**Scanner 1[a]**									
Diffusion	Axial DTI<br>Single shot spin echo EPI<br>15 -directions<br>B700	5500	minimum	NA	NA	NA	250	2.0/0	24.0
T1-weighted	3D SPGR	2	0	450	1	15	20.8	1/0	19.2
T2-weighted	3D CUBE	2000	100	NA	33	NA	50.00	1.00/0	22.4
SWI	SWAN	Minimum	Minimum	NA	NA	10	41.67	2.8/0	22.0
**Scanner 2[b]**									
Diffusion	Single shot spin echo EPI<br>b1000<br>SENSE acceleration	3000	75	NA	67	90	1500	4/0	20.0
T1-weighted	3D T1	9.57	5.47	NA	196	8	241	1/0	25.6
T2-weighted	Axial 2D T2	5038	100	NA	12	90	108	3/0	20.0
SWI	SWI<br>CS-SENSE acceleration	20	27	NA	NA	7	171	1.8/0	20.0

[a]*Scanner 1 was a GE Discovery 750 (3T).* [b]*Scanner 2 was a Philips Intera (1.5T). SWI, susceptibility weighted imaging. NA, not applicable.*

Supplemental Table 2. Sensitivity Analyses, Association of White Matter Injury Severity on Ultrasound with White Matter Injury Severity on MRI

	**White Matter Injury Severity, Term Equivalent Age MRI**			
	Excluding severe intraventricular hemorrhage[a]		Excluding cystic white matter injury[b]	
Predictor	OR (95% C.I.)	p-value	OR (95% C.I.)	p-value
Univariate				
7 DOL ultrasound	1.6 (1.1-2.3)	**0.02**	2.5 (1.6-3.9)	**<0.01**
30 DOL ultrasound	1.6 (1.2-2.2)	**<0.01**	1.8 (1.1-3.0)	**0.03**
Maximum ultrasound grade	1.5 (1.1-2.1)	**<0.01**	1.8 (1.1-2.9)	**0.01**
Multivariable[c]				
7 DOL ultrasound	1.7 (1.1-2.5)	**0.01**	2.6 (1.7-4.3)	**<0.01**
30 DOL ultrasound	1.5 (1.1-2.1)	**0.01**	1.7 (1.0-3.0)	0.08
Maximum ultrasound grade	1.5 (1.1-2.0)	**0.02**	1.8 (1.1-3.2)	**0.04**

Ordinal logistic regression was used to determine the association between the grade of white matter injury on ultrasound (7 days of life [DOL], 30 DOL, or "maximum" head ultrasound representing the higher score between 7 and 30 DOL) and grade of white matter injury on term equivalent age MRI. OR, odds ratio. C.I., confidence interval. [a]Patients with grade 3 germinal matrix hemorrhage/intraventricular hemorrhage or periventricular hemorrhagic infarction were removed for this analysis, resulting in N=39 infants. [b]Patients with cystic injury on head ultrasound (grade 5) were removed for this analysis, resulting in N=44 infants. [c]Multivariable models were adjusted for gestational age at birth and IVH severity on the same scan (7 DOL HUS, 30 DOL HUS, maximum ultrasound grade, or term equivalent age MRI).
