# Supplementary material for: Early ultrasound-based assessment of preterm white matter injury: association with MRI and neurological outcomes
Source: Pediatr Radiol. 2026 Feb 18;56(6):1308–19. doi: 10.1007/s00247-026-06528-y (PMC13212370; doi:10.1007/s00247-026-06528-y)
Supplement: Supplementary file 2 — (75.9 KB PDF) [file 247_2026_6528_MOESM2_ESM.pdf]

Supplementary Methods

Project Design, Setting, and Participants

Based on our institution's neuroimaging screening guidelines, all neonates born at equal to or less than 32 weeks gestational age (GA) receive 7 and 30 day of life (DOL) head ultrasounds. Patients are recommended to receive a term-equivalent age MRI if head ultrasound abnormalities are present, if GA at birth is less than 28 weeks, or if the clinician determines that the patient is "high risk" for brain injury, secondary to clinical factors including a history of meningitis, sepsis, or severe lung disease. The sample size of 50 subjects was determined by practical limitations (availability of imaging studies performed on eligible patients), not by an a priori power analysis. An electronic health record search for preterm infants born at ≤ 32 weeks completed gestation between 2015 and 2023 resulted in 222 patients who underwent the same imaging studies (7 and 30 DOL head ultrasound and term-equivalent age brain MRI). Our cohort therefore represented 22.5% (50/222) of premature infants who underwent the same brain imaging screening protocol during the same timeframe at our institution. This study was exempt from a requirement for patient consent.

Neurological Outcomes

In accordance with our institution's standard clinical practice, all neonates born at ≤ 32 weeks GA are referred to a High Risk Infant Follow-Up clinic after discharge for routine neurodevelopmental follow-up. Many patients also are referred for follow-up in pediatric neurology, audiology, and/or ophthalmology clinics. Out of the 50 infants included in the study, 1 was lost to follow-up and 1 was found to have Noonan Syndrome and thus was excluded from analysis of neurodevelopmental outcomes. Neurological outcomes were assessed in the remaining 48 subjects. Cerebral palsy (CP) was defined as a fixed neurologic deficit or movement disorder attributed to non-progressive

disturbances that occurred in the developing fetal or infant brain [1, 2]. Vision impairment was defined as complete vision loss or an impairment requiring corrective lenses or surgery. Hearing impairment was defined as any sensorineural hearing loss, conductive hearing loss, or hearing loss requiring hearing aids. Epilepsy was defined as two unprovoked seizures twenty-four hours apart, or one unprovoked seizure with a high possibility of seizure recurrence (due to, for example, an abnormal EEG and/or brain MRI).

Reference for Supplemental Methods

1. Fisher RS, Acevedo C, Arzimanoglou A, et al (2014) ILAE Official Report: A practical clinical definition of epilepsy. Epilepsia 55:475–482. https://doi.org/10.1111/epi.12550

2. (2007) The Definition and Classification of Cerebral Palsy. Dev Med Child Neurol 49:1–44. https://doi.org/10.1111/j.1469-8749.2007.00001.x
